# A Polysaccharide-Based Oral-Vaccine Delivery System and Adjuvant for the Influenza Virus Vaccine

**DOI:** 10.3390/vaccines12101121

**Published:** 2024-09-29

**Authors:** Chaitanya K. Valiveti, Mrigendra Rajput, Neelu Thakur, Tooba Momin, Malabika Bhowmik, Hemachand Tummala

**Affiliations:** 1 Department of Pharmaceutical Sciences, College of Pharmacy & Allied Health Professions, South Dakota State University, Brookings, SD 57007, USA; chaitanya.valiveti@jacks.sdstate.edu (C.K.V.); hemachand.tummala@sdstate.edu (H.T.); 2 Department of Biology, University of Dayton, Dayton, OH 45469, USA; nthakur1@udayton.edu (N.T.); momint1@udayton.edu (T.M.); bhowmikm1@udayton.edu (M.B.)

**Keywords:** influenza virus vaccine, inulin acetate, mucosal vaccine, influenza A nucleoprotein, subunit vaccine

## Abstract

Influenza virus enters the host body through the mucosal surface of the respiratory tract. An efficient immune response at the mucosal site can interfere with virus entry and prevent infection. However, formulating oral vaccines and eliciting an effective mucosal immune response including at respiratory mucosa presents numerous challenges including the potential degradation of antigens by acidic gastric fluids and the risk of antigen dilution and dispersion over a large surface area of the gut, resulting in minimal antigen uptake by the immune cells. Additionally, oral mucosal vaccines have to overcome immune tolerance in the gut. To address the above challenges, in the current study, we evaluated inulin acetate (InAc) nanoparticles (NPs) as a vaccine adjuvant and antigen delivery system for oral influenza vaccines. InAc was developed as the first polysaccharide polymer-based TLR4 agonist; when tailored as a nanoparticulate vaccine delivery system, it enhanced antigen delivery to dendritic cells and induced a strong cellular and humoral immune response. This study compared the efficacy of InAc-NPs as a delivery system for oral vaccines with Poly (lactic-co-glycolic acid) (PLGA) NPs, utilizing influenza A nucleoprotein (Inf-A) as an antigen. InAc-NPs effectively protected the encapsulated antigen in both simulated gastric (pH 1.1) and intestinal fluids (pH 6.8). Moreover, InAc-NPs facilitated enhanced antigen delivery to macrophages, compared to PLGA-NPs. Oral vaccination studies in Balb/c mice revealed that InAc-Inf-A NPs significantly boosted the levels of Influenza virus-specific IgG and IgA in serum, as well as total and virus-specific IgA in the intestines and lungs. Furthermore, mice vaccinated with InAc-Inf-A-NPs exhibited notably higher hemagglutination inhibition (HI) titers at mucosal sites compared to those receiving the antigen alone. Overall, our study underscores the efficacy of InAc-NPs in safeguarding vaccine antigens post-oral administration, enhancing antigen delivery to antigen-presenting cells, and eliciting higher virus-neutralizing antibodies at mucosal sites following vaccination.

## 1. Introduction

The mucosal surface covers approximately 400 square meters of areas in adult human beings, including the lining of the gastrointestinal, genitourinary, and respiratory tracts, the surface of the eyes, and the ear canal [1,2,3]. These mucosal linings frequently encounter pathogens and provide an entry path for >90% of known pathogens, including a variety of viruses such as influenza and SARS-CoV-2 [4,5,6,7,8,9,10]. Efficient immune response at mucosal sites can prevent those diseases. Traditional vaccine administration via parental routes elicits a strong systemic immune response but often falls short of generating effective mucosal immunity [11]. In contrast, vaccines delivered through the mucosal route have the potential to induce a strong mucosal immune response characterized by the production of virus-neutralizing secretory antibodies, notably immunoglobulin A (sIgA), and the activation of immune cells [4,11].

Formulating mucosal vaccines, such as oral vaccines is very challenging mainly due to the destruction of antigen and delivery system by the low pH gastric fluids with digestive enzymes as well as properties of the gut to develop immune tolerance for orally administered antigen [12,13]. Furthermore, vaccine antigens must reach the gut-associated lymphoid tissue (GALT) while overcoming physical and chemical barriers that hinder their internalization [14]. The dilution of vaccine antigens by large volumes of food and fluid necessitates higher antigen doses to evoke an immune response compared to traditional parenteral immunizations [15]. These challenges are further amplified for subunit vaccines due to their poor immunogenicity despite several advantages they offer such as better safety, the potential to combine multiple epitopes from several proteins, and the ease of modifying the epitopes based on the evolving variant [6,7,8,9,10,11,12,13,14,15,16,17,18].

Three strategic approaches combined could address the above challenges of oral vaccine development: (a) entrapping antigens in a gastric-resistant matrix to shield them from the harsh gastric environment, (b) facilitating antigen uptake by specific regions or cells within the GALT, and (c) activating the immune cells using an adjuvant to break the tolerance.

In this study, we are testing the ability of Inulin acetate-based nanoparticles as an oral vaccine delivery system using influenza A nucleoprotein as an antigen that potentially covers all the proposed approaches. (a) InAc is a hydrophobically modified polymer of a plant polysaccharide inulin, which is resistant to gastric enzymes and acidic pH. (b) being a very stable hydrophobic particulate delivery system, it is expected to be taken up by GALT, and (c) InAc is established as a TLR4 agonist that activates antigen-presenting cells to stimulate a strong humoral and cell-mediated immune response against encapsulated antigen [2,19,20]. However, these previous studies have used InAc as an adjuvant through a systemic route to activate the immune system. In this study, for the first time, we have investigated the InAc nanoparticles (InAc-NPs) to deliver the encapsulated influenza-A nucleoprotein (Inf-A) antigen orally in mice and evaluated their ability to stimulate a mucosal immune response.

## 2. Materials and Methods

### 2.1. Materials

We purchased the Inulin (catalog number: 198971) and polyvinyl alcohol (catalog number 151937) from MP Biomedicals, Solon, OH, USA. While Poly (D, L-lactide-co-glycolide), Resomer^®^ RG 503 (catalog number 739952), egg albumin (catalog number A5503), and bovine serum albumin (catalog number A3059) were purchased from Sigma-Aldrich, St. Louis, MO, USA and Fluorescein isothiocyanate isomer I (catalog number SC-206015A) was purchased from Santa Cruz Biotechnology, Inc. Dallas, TX, USA. BD sheath additive (catalog number 660584), BD detergent solution concentrate (catalog number 660585), and BD FACS clean (catalog number 340345) were purchased from BD Biosciences, San Jose, CA, USA. All the other chemicals were purchased from Fisher Scientific, Waltham, MA, USA.

### 2.2. Cells and Animals

Wild-type mouse macrophages (catalog number NR-9456) were kindly provided by BEI Resources, ATCC, Manassas, VA, USA. Macrophages were cultured in DMEM-high glucose medium (catalog number, SH30022.FS Hyclone Laboratories, Logan, UT, USA) and supplemented with antibiotics (gentamycin and penicillin/streptomycin) (catalog number P7539, Sigma Aldrich, St Louis, MO, USA) and 10% fetal bovine serum (FBS) (catalog number 16140071, Thermo Fisher Scientific, Cincinnati, OH, USA). Balb/c mice used in this study were purchased from Taconic Biosciences, Cambridge, IN, USA.

### 2.3. InAc-Influenza A Nanoparticles (InAc-Inf-A-NPs) Formulation

Inf-A antigen [Influenza A nucleoprotein (NP), 366–374: Strain A/PR/8/35, RP20267, GenScript Biotech, Piscataway, NJ, USA] was encapsulated into InAc-NPs by double emulsion (*w*/*o*/*w*) solvent evaporation technique as standardized previously [21]. Briefly, Inf-A antigen dissolved in 10 mM phosphate buffer (pH 7.4) containing 2% Pluronic-F68, was emulsified with dichloromethane (DCM) containing InAc. The primary emulsion was then added to water containing 0.5% *w*/*v* PVA and stirred continuously for 14 h. The resulting precipitated particles were collected by centrifugation at 20,000× *g*, lyophilized with 20 mg of mannitol as a cryoprotectant, and stored at −20 °C until further use. Antigen-encapsulated poly(lactic-co-glycolic acid) (PLGA) nanoparticles (NPs) were prepared using a similar procedure.

### 2.4. Analysing the Morphology of InAc-Inf-A-NPs Using a Scanning Electron Microscope (SEM)

The morphology of InAc-Inf-A nanoparticles (NPs) was analyzed using scanning electron microscopy (SEM, Model S-3400N, Hitachi, Japan). Lyophilized InAc-Inf-A-NP powder was mounted on metal holders with conductive double-sided tape and sputter-coated with a 10 nm gold layer. Nanoparticle images were captured at an accelerating voltage of 5–25 kV, a working distance of 5–15 mm, and a spot size of 3, as previously described [2,21]. The particle diameters were then measured using ImageJ software (Version 1.54).

### 2.5. Analysing the Size and Charge of InAc-Inf-A-NPs Using Dynamic Light Scattering (DLS)

The size and zeta potential (charge) of InAc-Inf-A nanoparticles (NPs) were further analyzed using dynamic light scattering (DLS) with a Malvern Zeta-Sizer (Malvern Ltd., Westborough, MA, USA). In brief, 2 mg of InAc-Inf-A-NPs were dispersed in filter-sterilized deionized water. The particle size (nm) and zeta potential (mV) were measured and reported as percent intensity, as previously described [21].

### 2.6. Quantification of Encapsulated Influenza A Nucleoprotein (Inf-A Peptide) in InAc-Inf-A-NPs

To quantify the encapsulated Inf-A peptide in InAc-Inf-A-NPs, one mg of antigen-loaded NPs was added to the acetone/dimethylformamide (DMF) (500 µL) to dissolve the polymer. The resultant precipitated Inf-A peptide (antigen) was pelleted at 4 °C by centrifugation at 20,000× *g* for 15 min. The collected pellet was dissolved in 500 µL, 10 mM phosphate buffer, pH 7.4 containing 1% sodium lauryl sulfate. The Inf-A peptide concentrations in solution were determined via HPLC using a Waters HPLC system with a W2998 PDA detector and Eclipse XDB-C18 4.6 × 150 mm column. A gradient method (Table 1) with mobile phase-A (0.05% TFA in water) and mobile phase B (0.01% TFA in acetonitrile) at a constant flow rate of 0.7 mL/min, with 20 µL sample injection. The peptide concentration was detected at 220 nm wavelength.

A standard curve from known Inf-A peptide amounts (*n* = 3) was used to quantify unknown amounts in samples, reported as µg of antigen per mg of Inf-A-NPs.

### 2.7. Determining the Stability of InAc-NPs in Gastric Fluids

The InAc-NPs loaded with fluorescein sodium dye (3 µg dye/mg of NP) (catalog number F6377, Sigma Aldrich, St Louis, MO, USA) were washed three times with PBS with 10 min of incubation between each wash to remove surface adsorbed fluorescein sodium. After washing, the NPs were dispersed in three different media: deionized water, simulated gastric fluid (SGF), pH: 1.1 (catalog number 7108, Ricca Chemical, Pocomoke City, MD, USA), and simulated intestinal fluid (SIF), pH: 6.8 (catalog number R7109100, Ricca Chemical, Pocomoke City, MD, USA). The dispersed samples were incubated in an orbital shaker at a speed of 100 rpm at 37 °C for 24 h, and samples were collected at 0, 5, 10, 15, 20, and 24 h. Samples were centrifuged at 20,000× *g* for 15 min, and the resulting supernatant was neutralized to pH 7.4 with phosphate buffer to minimize the quenching of fluorescein after release. Analysis of fluorescein sodium presence was conducted using a fluorimeter with excitation at 460 nm and emission at 515 nm. Fluorescein sodium concentration in the supernatant was determined by comparing its fluorescent intensity with 100% release from InAc-fluorescein sodium-NPs dissolved in acetone/DMF and diluted with phosphate buffer. A 24 h time point was selected as the maximum duration, considering typical food transit through the small intestine (~6 h post-ingestion) and passage through the colon (~60 h). Normal defecation typically occurs within 24 h [22].

### 2.8. Determining the Internalization of InAc-NPs by Murine Macrophages

To assess the impact of InAc-NPs on antigen delivery to antigen-presenting cells (APCs), we utilized FITC-labeled ovalbumin (FITC-Ova) as a tracer to monitor antigen uptake by macrophages. FITC-Ova was encapsulated within InAc-NPs and PLGA-NPs. Murine macrophage cells (1 × 10^6^/well) were seeded in a 24-well plate and treated with media alone (control), 25 µg of FITC-Ova encapsulated in InAc-NPs (InAc-FITC-Ova-NPs), or PLGA-NPs (PLGA-FITC-Ova-NPs) for 1 h at 37 °C. After treatment, cells were washed thrice, trypsinized to form a single-cell suspension, and analyzed for the percentage of cells containing FITC-Ova and the mean fluorescent intensity (MFI) per cell via flow cytometry (Becton-Dickson, Mountain View, CA, USA).

### 2.9. Analyzing the Efficacy of InAc-Inf-A-NPs as a Vaccine Adjuvant and an Oral Vaccine Delivery System in Stimulating an Immune Response

The effect of InAc-NPs as a vaccine adjuvant and oral vaccine delivery system in inducing an immune response specific to Influenza A was investigated using Balb/c mice. Fifteen, ten-week-old male and female Balb/c mice were obtained from Taconic Biosciences, Cambridge, IN, USA. Upon arrival, the mice were divided into three groups, with three males and two females in each group, and allowed to acclimatize to laboratory conditions for one week. After this acclimatization period and overnight fasting, mice were orally fed with the following treatments: 50 µL saline (group 1), 100 µg Influenza A peptide (Influenza A NP, 366–374 aa, Strain A/PR/8/35 peptide, GeneScript, Pennington, NJ, USA) in total volume of 50 µL of saline (group 2), or InAc-Inf-A-NPs containing 100 µg Influenza A peptide with total volume of 50 µL in saline (group 3).

After one week of the initial oral vaccination, all mice were subjected to oral booster doses. Blood samples were collected from the tail vein on day 0 (before vaccination), one week after the first dose, and five weeks after the first dose. All the mice were sacrificed five weeks after the first vaccination, and organs such as the intestine, lung, and spleen were harvested and snap-frozen in liquid nitrogen for analysis of tissue-specific IgA titer. All animal procedures were performed in compliance with the approved IACUC protocol (RAJPUT_091019) at Arkansas Tech University, Arkansas.

### 2.10. Quantifying the Concentrations of Influenza A-Specific IgG and IgA in Serum Samples

Blood samples were collected at 0 days, one week (Day 7), and five weeks (Day 35) post-first vaccination. Collected blood samples were used to separate serum through centrifugation at 1000× *g* for 15 min at 4 °C. The isolated serum samples were analyzed for Influenza A-specific IgG concentrations using the Influenza A Virus NP Antibody Inhibition ELISA (Virusys Corporation, Milford, MD, USA) according to the manufacturer’s protocol. Then, the Nucleoprotein Reduction Index (NPRI) for each sample was converted into a fold change in Influenza A-specific IgG concentration as compared to the control group (e.g., mice treated with saline).

For the measurement of Influenza A-specific serum IgA concentration, the IgA Mouse Uncoated ELISA Kit (Invitrogen, Waltham, MA, USA) was used with slight modifications. The ELISA plates were coated with the Influenza A peptide (strain A/PR/8/35, amino acids 366–374) which was purchased from GeneScript, Piscataway, NJ, USA. Influenza A peptide was dissolved in 1X coating buffer to achieve a final concentration of 0.4 mg/mL. A 100 µL of diluted peptide was added to each well, and the plates were incubated at 4 °C overnight. After overnight incubation, the plates were washed and blocked with washing and blocking buffer, supplied with the kit. The collected serum sample and IgA standards were diluted in assay buffer and added to the designated wells. Assay buffer was used in control wells to measure the background and plates were incubated for 2 h at room temperature. Plates were then washed four times before adding the detection antibodies to each well. After adding detection antibodies, plates were further incubated for 1 h at room temperature, then the plates were washed four times, and 100 µL of substrate was added to each well. The reaction was stopped with the addition of 100 µL of stopping solution, and the optical density (OD) was measured at 450 nm. The sample OD values were converted into IgA concentrations using a standard curve generated from serially diluted IgA standards and their corresponding OD values.

### 2.11. Quantifying the Total IgA and Influenza A-Specific IgA Concentrations in Tissue Samples from the Small Intestine (Ileum), Lungs, and Spleen

For measuring the total and Influenza A-specific IgA concentrations in tissue samples, the distal part of the ileum (a 2-inch segment from the ileocecal junction), all lobes of the lungs, and both spleens were collected at fifth weeks after the first vaccination. Collected tissue samples were immediately snap-frozen in liquid nitrogen after collection and stored at −80 °C until analysis. During IgA analysis, the frozen tissues were weighed and homogenized in phosphate-buffered saline (1X PBS, pH 7.4) to achieve a final tissue concentration of 200 mg/mL. Tissue homogenates were centrifuged at 4 °C, 8000 rpm for 3 min. The supernatants from tissue homogenates were collected and measured for total and virus-specific IgA concentrations. An IgA-Mouse Uncoated ELISA Kit (Invitrogen, Waltham, MA, USA) was used to measure the total IgA concentrations in the tissue supernatants according to the manufacturer’s protocol. For measuring Influenza A-specific IgA, an uncoated ELISA Kit (Invitrogen, Waltham, MA, USA) was used with slight modification as mentioned above and converted the units to IgA/gram of tissue.

### 2.12. Hemagglutination Inhibition Assay (HI Assay) to Measure Influenza-A-Specific Antibodies in the Ileum and Lungs

We used a hemagglutination inhibition (HI) assay to measure the Influenza A virus-neutralizing antibodies in the tissue supernatants as per the method previously described [23]. The Influenza A virus (strain A/Puerto Rico/8-9NMC1/1934: H1N1, provided by BEI Resources, NR-29023) was diluted in PBS to achieve a final concentration of 4 hemagglutination (HA) units in 25 µL. A 25 µL of tissue homogenate supernatant was two-fold serially diluted using 1X PBS in a 96-well round-bottom plate. While 1X PBS was used as a negative control. A 25 µL Influenza A virus (containing 4 HA units) was added to each well, and the plate was incubated for 30 min at room temperature to allow for virus neutralization. After 30 min of incubation, 1% chicken red blood cells (Innovative Research Inc, Novi, MI, USA) were added to each well and incubated for an additional 30 min. The hemagglutination inhibition (HI) unit for each sample was determined by identifying the highest dilution of tissue homogenate that exhibited a “button” formation, indicating successful virus neutralization by the Influenza A-specific antibodies.

### 2.13. Statistical Analysis

A Student’s *t*-test and one-way ANOVA followed by Tukey’s Honestly Significant Difference (HSD) Test at the 95% confidence level (e.g., *p* < 0.05 for Student’s *t*-test and α = 0.05 for one-way ANOVA) were used to evaluate the statistical significance of the InAc-NPs vaccine formulation’s effect on antigen delivery, immune activation, and the induction of Influenza A-specific immune responses.

## 3. Results

### 3.1. Physicochemical Characterization of the InAc Polymer and Vaccine Formulation

**Figure 1:**
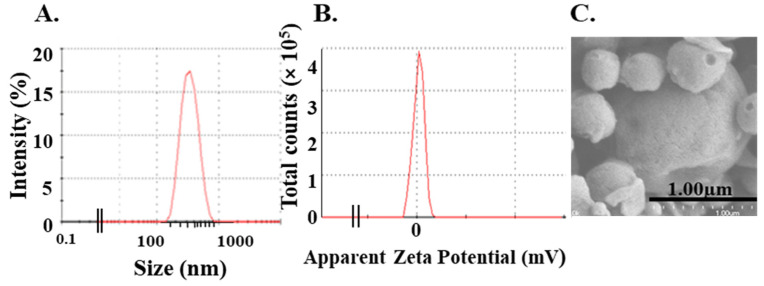
Characterization of InAc-Inf-A-NPs: (**A**) the mean particle size distribution was measured using DLS; (**B**) Zeta potential shows the surface charge of InAc-Inf-A-NPs a slightly negative or neutral (−0.9 ± 0.2 mV); (**C**) the morphology of InAc-Inf-A-NPs were spherical particles with a diameter of ~500 nm as shown by scanning electron microscopy (SEM).

In this study, we encapsulated Influenza A peptide (nucleoprotein from Influenza strain A/PR/8/35) as the antigen into InAc-NPs, serving both as a vaccine adjuvant and delivery system. These vaccine particles were labeled as InAc-Inf-A-NPs. The mean diameter of the InAc-Inf-A-NPs was 515 ± 0.86 nm, with a slightly negative surface charge of −0.9 ± 0.21 mV, as determined using DLS analysis (Figure 1A,B). The shape and the size were also examined using SEM, revealing InAc-Inf-A-NPs as spherical nanoparticles with ~500 nm in diameter (Figure 1C) confirming the size detected by the DLS method. The antigen (Inf-A) content was around 15.43 ± 0.7 µg per mg of InAc-Inf-A-NPs as determined by RP-HPLC.

### 3.2. InAc-NPs: A Promising Nano-Delivery System for Oral Vaccine Delivery

Oral vaccines offer several advantages over traditional parenteral vaccines. They eliminate the need for needles, simplifying administration, especially in large-scale vaccination programs. Oral vaccines also reduce needle-associated pain and anxiety while lowering the risk of needle-associated injuries and disease transmission. Specifically, nanoparticle-based oral vaccines can encapsulate fragile antigens, protecting them from degradation in the harsh gastrointestinal environment. This encapsulation facilitates precise antigen delivery to targeted cells, such as antigen-presenting cells, thereby enhancing the vaccine response.

**Figure 2:**
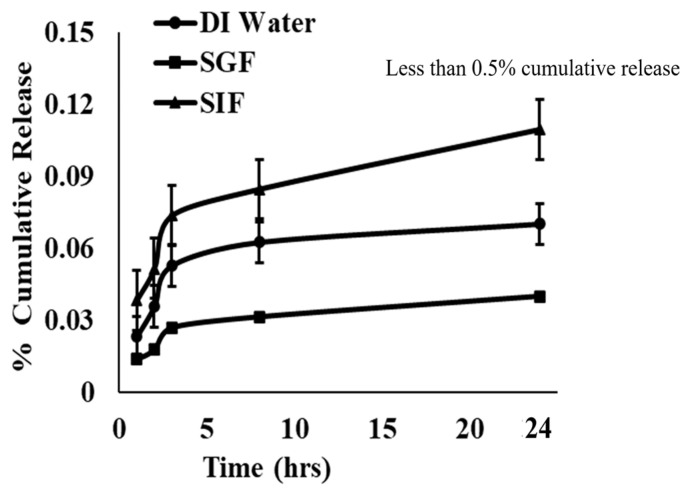
Efficacy of InAc-NPs in preventing premature release of the encapsulated antigen. InAc-NPs containing Fluoresceine Sodium dye as the encapsulated antigen were dispersed in DI Water, Simulated Gastric Fluid (SGF), or Simulated Intestinal Fluid (SIF). Suspension was incubated in an orbital shaker at a speed of 100 rpm at 37 °C for 24 h. Fluorescein concentration in the supernatant solution at different time points was measured by fluorimeter and % cumulated release was calculated by comparing its fluorescent intensity with 100% release of Fluoresceine Sodium from NPs dissolved in 100% acetone or DMF.

In the current study, we used fluorescein sodium as a model antigen to investigate whether InAc-NPs protect encapsulated antigens and prevent their premature release in gastric or intestinal environments. To execute this, fluorescein sodium was encapsulated within InAc-NPs at a concentration of 3 µg dye per milligram of nanoparticles as described in Section 2. Although oral vaccines are usually exposed to gastric fluid for 1–3 h and to intestinal fluids for 6–24 h, the NPs were incubated with both SGF and SIF for 24 h. Our results showed that InAc-NPs prevented the premature release of encapsulated dye for at least 24 h in both SGF and SIF. After 24 h of incubation, only a total of 0.8 ± 0.03%, 0.1 ± 0.00%, and 0.04 ± 0.00% of encapsulated fluorescein sodium was released in deionized water, SIF, and SGF, respectively (Figure 2). The release data suggests that InAc-NPs are stable in harsh gastrointestinal conditions and effectively protect encapsulated antigen(s) and prevent their release.

### 3.3. InAc-NPs Enhanced the Internalization of Antigens by Murine Macrophages

The uptake of antigens by antigen-presenting cells (APCs) like macrophages plays an important role in initiating and enhancing the immune response [24]. Earlier studies showed a direct relationship between the quantity of antigen presented to T cells and the activation of both, T cells and B cells [25,26].

**Figure 3:**
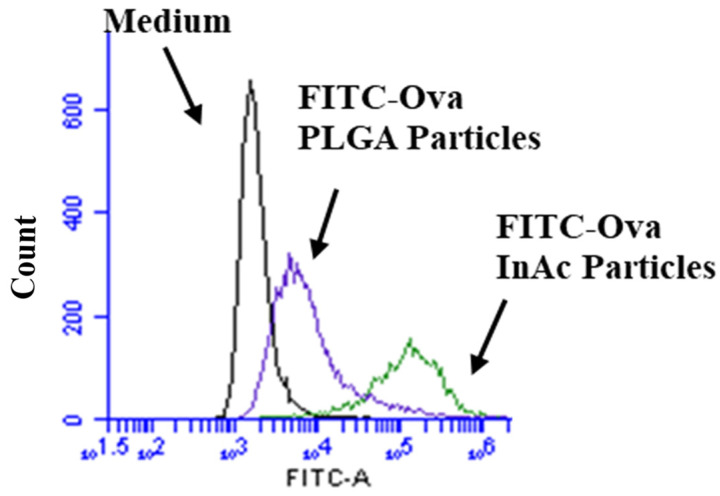
InAc-FITC-Ova-NPs uptake by murine macrophages. The InAc-FITC-Ova-NPs or PLGA-FITC-Ova-NPs each with 25 µg equivalent to FITC-Ova were incubated with wild-type macrophages. After 1 h incubation, the cells were analyzed by flow cytometry for the number of cells having the antigen (FITC-Ova, green fluorescence) and the relative amount of antigen per cell by mean fluorescent intensity (MFI).

To evaluate the efficacy of InAc-NPs in delivering encapsulated antigens to macrophages, wild-type macrophages were incubated with a model antigen FITC-labelled ovalbumin (FITC-Ova) delivered through either PLGA- or InAc-NPs. A similar amount of antigen was loaded between PLGA and InAc-NPs (~16.0–18.0 µg FITC-Ova/mg of NPs) determined as shown previously [2]. To ensure equal exposure, macrophages were treated with either InAc-NPs or PLGA-NPs, each containing 25 µg of FITC-Ova, whereas macrophages treated without any formulation were used as the control (medium alone). As shown in Figure 3, antigens delivered through InAc-NPs significantly enhanced antigen uptake by macrophage. There was a higher proportion of macrophages with antigen (99.80 ± 0.05%) when antigen was delivered using InAc-NPs as compared to PLGA-NPs (84.19 ± 4.20%) (*p* < 0.05) (Figure 3).

Further, macrophages treated with InAc-FITC-Ova-NPs had ~7 times more antigen per cell [Mean fluorescence intensity (MFI): 160,497.5 ± 17,382.03] as compared to macrophages treated with PLGA-FITC-Ova-NPs (MFI: 21,828.27 ± 2018.09) (Table 2) (*p* < 0.05).

These findings suggested that InAc-NPs have a high efficacy in enhancing antigen delivery and promoting phagocytosis by macrophages.

### 3.4. InAc-Inf-A-NPs Induced a Strong Antigen-Specific Antibody Response in Serum

**Figure 4:**
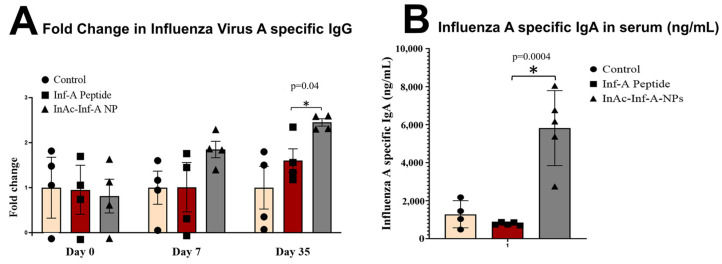
Fold change in Inf-A specific IgG (panel (**A**)) and IgA (panel (**B**)) in the serum following oral vaccination. BALB/c mice were vaccinated by oral administration of saline, Influenza A peptide alone in saline, or Influenza A peptide encapsulated in InAc-NPs (InAc-Inf-A-NPs). Two doses were given at one-week intervals. Blood was collected on day 0, day 7, and day 35 post-first vaccination. Panel (**A**) shows fold change in Inf-A-specific IgG tier at day 0, day 7-, and 35 days post-first vaccination while Panel (**B**) shows fold change in Inf-A-specific IgA tiers in serum at 35 days post-first vaccination. * Shows a significant difference at a 95% level of significance (*p* < 0.05).

Studies have shown that particulate antigens induce a stronger immune response as compared to soluble antigens [27,28]. In the current study, we converted the soluble Influenza A nucleoprotein into particulate antigen by encapsulating it in InAc-InfA-NPs for oral vaccine delivery. We further evaluated the effect of our vaccine formulation (e.g., InAc-Inf-A-NPs) on serum immunoglobulin G (IgG) and immunoglobulin A (IgA) titer in mice vaccinated with either 50 µL saline (control), 50 µL saline containing 100 µg of Influenza A peptide, or InAc-Inf-A-NPs containing 100 µg of Influenza A peptide. Our results showed that when mice were immunized with peptide alone in saline, there was no significant enhancement in the influenza A-specific IgG titers in the serum 35 days after the first vaccination. In contrast, delivering the same peptide using InAc-NPs produced a strong antibody titer, a 2.44 ± 0.05-fold increase in IgG titers compared to the unformulated influenza A peptide vaccine (*p* < 0.05) (Figure 4A). Similarly, influenza A-specific IgA levels in serum were significantly higher in mice vaccinated with InAc-Inf-A-NPs, which was reported as 5816.63 ± 1976.31 ng/mL, as compared to mice immunized with the influenza A peptide alone (780.50 ± 37.10 ng/mL) at 35 days post-vaccination (*p* < 0.05) (Figure 4B), around a 7.5-fold increase. Taken together, the data suggested that InAc-NPs as a delivery system not only protected antigens from premature release and degradation in the gastric environment but also delivered antigens to APCs to generate a strong humoral response.

### 3.5. InAc-Inf-A-NPs Induced a Strong Secretory (sIgA) Antibody Response in the Intestine and Lungs

A strong pathogen-specific immune response at mucosal sites plays a key role in preventing infections. Data from Figure 4 indicated that there is a strong production of antigen-specific IgG and IgA in the serum of mice immunized with InAc-Inf-A-NPs. In this study, we also investigated the effect of our vaccine formulation (e.g., InAc-Inf-A-NPs) on tissue-specific immunity by analyzing antibody concentration in the intestines, lungs, and spleen at 35 days post-first vaccination.

These tissue samples were assessed for total and Influenza A-specific immunoglobulin A (IgA) concentrations. The results indicated that the total IgA concentrations in both the intestines and lungs were markedly elevated after oral administration of InAc-Inf-A-NPs, as compared to the oral administration of influenza A peptide alone.

**Figure 5:**
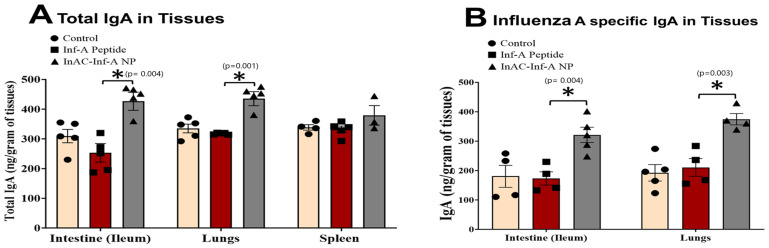
The concentration of total IgA (panel (**A**)) and Inf-A specific IgA (panel (**B**)) in the tissues following oral vaccination. BALB/c mice were orally vaccinated with two doses of saline, Influenza A peptide alone in saline, or InAc-Inf-A-NPs one week apart. Following five weeks of the first vaccination, the mice were sacrificed, and the tissues such as ileum (small intestine), lungs, and spleen were collected. Collected tissue samples were homogenized in protease inhibitor and normalized for equal protein concentration followed by measuring the concentration of total IgA (panel (**A**)) and influenza virus A specific IgA (panel (**B**)) by sandwich ELISA. * shows a significant difference at a 95% level of significance (*p* < 0.05).

In the intestine, the total IgA concentration was significantly higher in the mice vaccinated with InAc-Inf-A-NPs, measuring 426.70 ± 30.42 ng/g, compared to 253.14 ± 31.21 ng/g in mice treated with unformulated peptide. Similarly, in the lungs, the InAc-Inf-A-NPs group showed a total IgA concentration of 435.29 ± 23.70 ng/g, which was significantly higher than the 317.41 ± 1.34 ng/g observed in the peptide-only group (Figure 5A). However, there was no significant difference in total IgA concentration in the spleen between the InAc-Inf-A-NPs group (379.07 ± 25.61 ng/g) and the peptide-only group (332.30 ± 10.96 ng/g) (Figure 5A).

Similar to total antibody levels, Influenza A virus-specific IgA concentrations were significantly higher in both the intestines and lungs following oral vaccination with InAc-Inf-A-NPs vs. vaccination with peptide in saline. In the intestines, the InAc-Inf-A-NPs treatment resulted in a virus-specific IgA concentration of 321.22 ± 35.37 ng/g, significantly higher than the 173.14 ± 22.53 ng/g measured with the peptide alone (Figure 5B, *p* < 0.05). Similarly, in the lungs, the InAc-Inf-A-NPs treatment produced a virus-specific IgA concentration of 374.67 ± 40.09 ng/g, which was significantly greater than the 210.67 ± 8.98 ng/g observed with the peptide-only treatment (*p* < 0.05) (Figure 5B). These findings suggest that the InAc-Inf-A-NPs formulation significantly enhances the mucosal immune response in the intestines and lungs compared to traditional peptide-based vaccines, potentially leading to improved protection against respiratory pathogens such as Influenza A.

### 3.6. InAc-Inf-A-NPs Significantly Enhanced Virus-Specific Hemagglutination Inhibition (HI) Titer in Lungs and Intestine

Although neutralizing antibody titers provides reliable measures of antibody-mediated protection, studies have shown that HI titer is a very sensitive method in detecting influenza virus-neutralizing antibodies. There is a direct and close relationship between HI and virus neutralization test (NT) across different straining and subtypes. The HI assay is considered the gold standard method for assessing the effectiveness of influenza vaccines by measuring virus-neutralizing antibodies [29,30]. In the current study, the presence of virus-specific neutralizing antibodies in tissue homogenates, such as those from the lungs and intestines, was further confirmed through a HI assay. Our results revealed that the InAc-Inf-A-NPs vaccine significantly enhanced HI titers in both the intestines and the lungs (*p* < 0.05).

**Figure 6:**
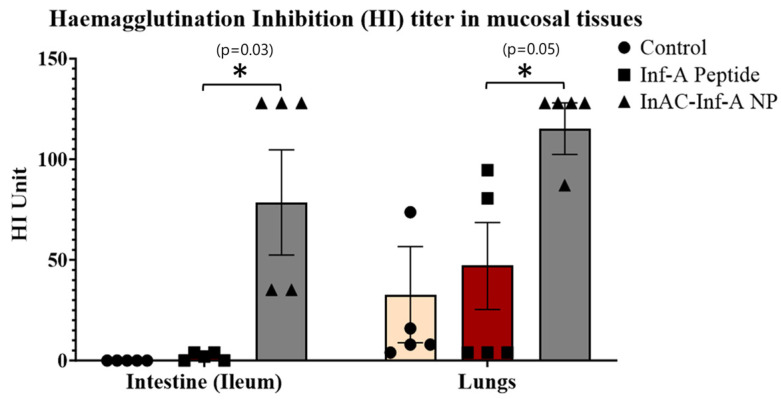
Hemagglutination inhibition (HI) titer following oral vaccination. BALB/c mice were orally vaccinated with two doses of saline, Influenza A peptide alone in saline, or InAc-Inf-A-NPs one week apart. After five weeks of the first vaccination, mice were sacrificed, and tissues were collected. The tissue samples were homogenized in protease inhibitor and supernatants of these homogenates were analyzed for the functionality of Influenza A virus-specific antibodies using HI assays. * shows a significant difference at a 95% level of significance (*p* < 0.05 in HI titer in tissue homogenates.

In the intestines, mice vaccinated with InAc-Inf-A-NPs exhibited significantly higher HI titer, with an average value of 78.40 ± 30.37, compared to mice vaccinated with saline (0.00 ± 0.00) and those vaccinated with Influenza A peptide alone (2.00 ± 0.89). Similarly, in the lungs, the HI titer for the InAc-Inf-A-NPs group was 115.20 ± 12.80, which was significantly higher than the saline group (32.80 ± 23.88) and the group vaccinated with Influenza A peptide alone (47.20 ± 26.93) (*p* < 0.05) (Figure 6).

## 4. Discussion

Previously, we determined the efficacy of InAc-NPs in enhancing the immune response of a parenteral vaccine [1]. The current work is carried out to evaluate the efficacy of InAc-NPs as an oral vaccine adjuvant and antigen delivery system in inducing a higher immune response. Needle-free vaccinations, such as oral vaccines, offer multiple benefits over traditional injection-based vaccines, which include reduced pain and discomfort, a lower risk of needle-related infections, and oral vaccines induce both systemic and mucosal immune response including efficient post-vaccination immune response in gut-associated lymphoid tissue (GALT) at mucous membranes of the gastrointestinal tract, with easy administration [31,32]. Oral vaccines encounter multiple challenges, such as the necessity for formulations that protect antigens from the harsh conditions of proteolytic enzymes and the extremely low pH that is present in the gastrointestinal tract [13], development of immune tolerance to orally administered antigens and antigens may become diluted, and dispersed in food and mucosal secretions [14]. The dispersion of vaccine formulations in mucosal secretions creates an additional barrier to their delivery to immune cells. The results from this manuscript indicated that InAc-NPs effectively protected the antigen from harsh environments like simulated gastric fluid (pH 1.1) and simulated intestinal fluid (pH 6.8), maintaining stability for up to 24 h. Gastric emptying, transit through the small intestine, and food passage to the colon depend on factors like age, health, and food type [33,34]. Under normal conditions, food can transit through the small intestine within 6 h post-ingestion, where most intestinal immune cells are located. However, in some cases, food can remain in the colon for about 60 h [22]. We selected a 24 h duration to measure the efficacy of InAc-NPs in protecting the antigen, as this timeframe is typically enough for an oral vaccine to reach Peyer’s patches, a key region in the small intestine where immune responses are initiated [35]. InAc-NPs were prepared with a water-insoluble polymer inulin acetate, a derivative of inulin [21]. Inulin acetate is water-insoluble, and its backbone structure has modified poly-fructose with beta (2→1) linkages in linear chains that are resistant to digestion by gastric enzymes and acidic conditions. Additionally, it has excellent quality for preparing nanoparticles (NP) and encapsulating water-soluble proteins/antigens [1]. Hence, it is expected that it will not release its encapsulated antigens within the gastrointestinal (GI) transit time such as in the stomach [36,37]. Therefore, as anticipated, only less than 1% of antigen is released from the InAc-NPs in 24 h, with around 99 percent of antigen retained inside the particles. The release data suggests that there is non-significant diffusion of the aqueous medium inside the InAc-NPs to dissolve and release water-soluble antigens. Taken together, the InAc-NPs delivery system could protect antigens in harsh gastric environments.

Once protected by the gastric environment, for vaccine formulations, it is critical to efficiently deliver antigen to antigen-presenting cells (APCs) to generate an effective immune response. Our results showed that InAc-FITC-Ova-NPs delivered the vaccine formulation to APCs (macrophages) more effectively than antigens encapsulated in PLGA nanoparticles (NPs) (e.g., PLGA-FITC-Ova-NPs). We used PLGA NPs to compare the antigen delivery efficiency of InAc-NPs, as PLGA-NPs are a well-established drug delivery system [38] and a platform for subunit vaccine delivery [39]. Our current study showed that InAc-NPs are superior to PLGA-NPs in delivering antigens to wild-type macrophages, potentially due to their recognition through TLR-4 receptors. Our previous study with macrophages from TLR-4 knockout and wild-type mice demonstrated that InAc-NPs act as a TLR4 agonist, as they significantly increased IL-6 and nitric oxide production in wild-type macrophages but failed to induce these responses in TLR-4-knockout macrophages [20]. Since TLR-4 plays a crucial role in activating immune cells and enhancing phagocytosis, this property of InAc-NPs may be responsible for having enhanced vaccine formation uptake by macrophage in the current study [20,40,41]. Additionally, the nanosized InAc particles which mimic the size and shape of viruses or pathogens may further help in increased phagocytic activity [42].

Currently, available Influenza virus vaccines protect by neutralizing antibodies against the virus surface glycoproteins hemagglutinin (HA) and neuraminidase (NA). However, because the HA and NA genes in influenza viruses frequently mutate, new variants emerge almost every year, allowing the virus to evade pre-existing immunity established by these vaccines [43]. Therefore, developing vaccines against more conserved antigens in the influenza virus could be beneficial in preventing seasonal influenza virus infections. Several peptide-based antigens, like transmembrane protein M2 and nucleoprotein (NP), have been evaluated for broader protection against a variety of influenza virus strains [44]. Vaccination with Influenza A nucleoprotein enhanced virus-specific CD8+ T-cell response to the influenza virus and stimulated the production of tumor necrosis factor-alpha (TNF-α) and antiviral cytokines like interferon-gamma (IFN-γ) [45,46]. Similarly, a DNA vaccine encoding the influenza virus nucleoprotein induced strong antibody and T-cell responses and protected against highly pathogenic influenza virus strains such as H5N1 [47], whereas, a mucosal vaccine containing nucleoprotein showed a broad protection against two distinct lineages of influenza B virus [48]. The ability of the influenza virus nucleoprotein to induce heterosubtypic immunity has made it a key candidate for protein-based vaccine trials. These trials have produced encouraging results, demonstrating higher virus-specific cellular responses and nucleoprotein-specific antibody responses, pointing toward its effectiveness in broader influenza protection [49,50]. In this study, we used a peptide sequence (amino acids 366–374) from the nucleoprotein of Influenza strain A/PR/8/35 as a vaccine antigen to evaluate the effectiveness of an influenza vaccine. However, peptide antigens like Influenza A nucleoprotein, tend to have low immunogenicity on their own and require vaccine adjuvants in the formulation to generate a better immune response [51].

The current study utilized Influenza A nucleoprotein as an antigen and InAc-NPs as a vaccine adjuvant and delivery system. Our results indicated that the InAc-Inf-A-NPs formulation significantly increased virus-specific IgG and IgA titer at 35 days post-first vaccination, suggesting an enhanced immune response compared to unformulated antigens. Serum IgA comprises approximately 15% of total serum immunoglobulin in healthy individuals [52,53,54] and antigen-specific serum IgA can be a useful indicator of mucosal immune response [55]. To determine the immune response of our vaccine formulation (InAc-Inf-A-NPs), we measured total and virus (antigen) specific IgA levels in serum and various organs, including the intestine, lungs, and spleen. Our results showed that InAc-Inf-A-NPs significantly increased total and virus-specific IgA titers in serum, intestine, and lungs, but not in the spleen. The lower virus-specific (antigen-specific) IgA in the spleen may be due to the predominance of IgA-producing plasma cells in mucosal membranes, such as those in the intestines, as compared to the spleen [56]. Earlier we explored the InAc-NPs as an intranasal antigen delivery system using ovalbumin (Ova) as a model antigen. After immunization with Ova-PBS, Ova encapsulated in PLGA-NPs or Ova encapsulated InAc-NPs, nasal-associated lymphoid tissue (NALT) was collected from mice at 42 days post 1st vaccination and analyzed antigen-specific IgA titer. Our results showed a 10-fold higher response in IgA titer in mice vaccinated with InAc-NPs compared to PLGA-NPs [20].

Virus-specific antibody titer provide a general indication of vaccine response, but virus-neutralizing antibodies are a more reliable marker for virus protection. In the case of the influenza virus, hemagglutination-inhibition (HI) titer are universally recognized as a gold standard method to measure protective immunity against the virus [57]. In the current study, we evaluated our formulation (InAc-Inf-A-NPs) in inducing Influenza A specific HI titer. Our results showed that InAc-Inf-A-NPs induced very strong Influenza A specific HI titers in serum, intestine, and lungs as compared to peptide alone. It would be interesting to find out how antibodies specific to the nucleoprotein of influenza A virus can neutralize the virus as indicated through higher HI titers. To explore the cross-reactivity of our antigen (e.g., Inf-A-nucleoprotein) specific antibodies with Inf-A-hemagglutinin (HA) or Inf-A-neuraminidase (NA), we obtained the protein sequence of the influenza A virus gene [Influenza A virus (A/Puerto Rico/8/1934(H1N1)] from the National Center for Biotechnology Information (NCBI) database, with GenBank ID: MH785011.1. We then analyzed the similarity between our antigen peptide, Inf-A-/PR/8/35- nucleoprotein, 366–374, and the protein sequences of Inf-A-HA or Inf-A-NA using the NCBI Protein BLAST tool [58]. Our analysis revealed no similarity between the Inf-A- nucleoprotein and Inf-A-NA protein sequences. However, Inf-A-nucleoprotein exhibited 80% identity with a sequence of five amino acids (NECME: Asparagine-Glutamate-Cysteine-Methionine-Glutamate) located at positions 490–494 in Inf-A-HA, and 60% similarity with the Inf-A-HA sequence SNASM (Serine-Asparagine-Alanine-Serine-Methionine) positioned at 285–289.

We further explored the role of these HA peptides, which showed similarity to our nucleoprotein antigen (e.g., Inf-A-nucleoprotein), in eliciting an antibody response and their reactivity to the antigen using the BepiPred-2.0: Sequential B-Cell Epitope Predictor software, with an epitope threshold of 0.53 [59,60]. This analysis revealed that the HA peptide sequence, which resembles the nucleoprotein, is part of a B-cell epitope. This indicates that the antibodies generated against our antigen peptides (e.g., Inf-A-nucleoprotein) could potentially cross-react with Inf-A-HA and contribute to virus neutralization, as reflected by the increased HI titer.

Overall, the current study assessed the ability of InAC-NPs as an oral vaccine delivery system for the very first time. Our study showed that InAC-NPs protected the vaccine antigen from degradation following oral administration it enhanced antigen delivery to antigen-presenting cells and induced a strong virus-neutralizing antibody at mucosal sites following vaccination.

## 5. Conclusions

Overall the current study demonstrated that InAC-NPs represent an effective vaccine adjuvant and delivery system for oral influenza vaccines. InAc-NPs not only protected the antigen from degradation in the harsh gastric and intestinal environments but also enhanced antigen uptake by macrophages and induced strong systemic and mucosal immune responses, both at the local sites in the intestine and distant mucosal sites in the lungs. Mice vaccinated with InAc-Inf-A-NPs showed significantly higher HI titers at mucosal sites, emphasizing their potential to elicit protective immunity at the entry point of the influenza virus. These findings suggest that InAc-NPs are a promising platform for the development of effective oral vaccines capable of overcoming the challenges associated with mucosal vaccination, providing an effective defense against influenza infection.

## Figures and Tables

**Table 1:** Gradient conditions for elution of Inf-A peptide. A total of 1 mg of antigen-loaded NPs was dissolved in 500 µL acetone/DMF to dissolve the polymer and release encapsulated Inf-A peptide (antigen). The released Inf-A peptide (antigen) was pelleted by centrifugation at 20,000× *g*, and the collected pellet was dissolved in 500 µL, 10 mM phosphate buffer, pH 7.4. Then, the concentrations of extracted Inf-A peptide in solution were determined by HPLC (Milford, MA, USA) with a W2998 PDA detector using the conditions as mentioned in the table.

Time (Minutes)	Flow Rate (mL/min)	Mobile Phase-A	Mobile Phase-B
0.0	0.7	80.0%	20.0%
5.0	0.7	25.0%	75.0%
10.0	0.7	80.0%	20.0%

**Table 2:** Quantification of antigen delivery to mouse macrophages. The InAc-FITC-Ova-NPs or PLGA-FITC-Ova-NPs, each with 25 µg equivalent to FITC-Ova were incubated with wild-type macrophages. After 1 h, the cells were analyzed by flow cytometry for the number of cells with green fluorescence/FITC-Ova. * and ** show a significant difference in the percent fluorescent positive cells compared to control cells (*) or PLGA-FITC-Ova-NPs treated cells (**), respectively, at 95% level of significance using one-way ANOVA followed by Bonferroni’s multiple comparison test.

S.No.	Treatment Groups	Mean Fluorescence Intensity
1.	Media	5678.48 ± 346.15
2.	PLGA-FITC-Ova-NPs	21,828.27 ± 2018.09 *
3.	InAc-FITC-Ova-NPs	160,497.5 ± 17,382.03 *^,^**

## Data Availability

The dataset is available from the corresponding authors upon reasonable request.
